# Changes in walking and running in patients with hip dysplasia

**DOI:** 10.3109/17453674.2013.792030

**Published:** 2013-05-31

**Authors:** Julie S Jacobsen, Dennis B Nielsen, Henrik Sørensen, Kjeld Søballe, Inger Mechlenburg

**Affiliations:** ^1^Department of Physiotherapy and Occupational Therapy, Aarhus University Hospital; ^2^Department of Sport Science, Aarhus University; ^3^Department of Orthopaedic Surgery, Aarhus University Hospital, Aarhus, Denmark.

## Abstract

**Background and purpose:**

Earlier studies have suggested that the hip extension angle and the hip flexor moment in walking are affected by hip dysplasia, but to our knowledge there have been no reports on running or evaluations of self-reported health. We evaluated differences in walking, running, and self-reported health between young adults with symptomatic hip dysplasia and healthy controls.

**Patients and methods:**

Walking and running in 32 patients with hip dysplasia, mean 34 (18–53) years old, was compared with walking and running in 32 controls, mean 33 (18–54) years old. Joint kinematics and kinetics—quantified by the peak hip extension angle and the peak net joint moment of hip flexion during walking and running—were recorded using a motion-capture system, and health was evaluated using the Copenhagen Hip and Groin Outcome Score (HAGOS).

**Results:**

The peak hip extension angle during walking was less in the patients than in the controls (–10.4 (SD 4.8) degrees vs. –13.2 (SD 4.5) degrees; p = 0.02). Similarly, the peak net joint moment of hip flexion during walking was lower in the patients than in the controls (0.57 (SD 0.13) N*m/kg vs. 0.70 (SD 0.22) N*m/kg; p = 0.008). In all dimensions of HAGOS, the patients scored lower than the controls. Furthermore, the hip extension angle and the net joint moment of hip flexion correlated with the HAGOS subscales pain and physical function in sport and recreation.

**Interpretation:**

Patients with symptomatic hip dysplasia do modify walking and running, and we therefore suggest that the impairment found in this study should play an important role in the evaluation of later operative and training interventions.

In developmental dysplasia of the hip, the acetabulum appears shallow and oblique with insufficient coverage of the femoral head (Anda et al. 1991, Klaue et al. 1991, Jacobsen et al. 2006). This leads to an increased load at the acetabular rim, and may initiate breakdown of the cartilage or labrum; eventually symptomatic osteoarthritis may develop (Cooperman et al. 1983, Murphy et al. 1995, Jacobsen and Sonne-Holm 2005).

Reduced hip extension angle, deficits of the hip flexors, and reduced walking velocity have been reported in patients with untreated hip dysplasia (Romano et al. 1996, Pedersen et al. 2004, Sucato et al. 2010). Apart from the reported hip deficits in the sagittal plane, an increased hip adduction angle and an increased external rotation angle together with changes to the net joint moments in the frontal and transversal plane were reported by Romano et al. (1996). Also, increased flexion of the trunk and increased anterior tilt of the pelvis could theoretically be present, with a reduction in the net joint moment of hip flexion (Saha et al. 2007, 2008, Chang et al. 2012). Previous studies have evaluated walking and standing only, but most of these young patients with hip dysplasia have higher expectations than just to be able to walk. There is a lack of information about high-intensity activities and specific patient-reported outcomes in patients with hip dysplasia.

We evaluated differences in walking, running, and self-reported health between young adults with symptomatic hip dysplasia and healthy controls. We hypothesized that patients with untreated hip dysplasia would present with a lower peak hip extension angle and a lower peak net joint moment of hip flexion in the sagittal plane than in healthy controls. Furthermore, we predicted that there would be correlations between the peak net joint moment of hip flexion and the peak hip extension angle on the one hand and the HAGOS subscales pain and physical function in sport and recreation (sport/recreation) on the other.

## Methods

### Participants

From March through December 2011, 32 patients with unilateral or bilateral hip dysplasia were included consecutively in the study (Figure). The patients were included from the Division of Hip Surgery of the Department of Orthopaedics at Aarhus University Hospital in Denmark. To be eligible, all patients had to meet the following criteria: (1) a diagnosis of hip dysplasia, (2) planned pelvis operation, (3) osteoarthritis of grade 0–1, and (4) age between 18 and 60 years. The exclusion criteria were: (1) hip dysplasia caused by Calvé Perthes or epiphysiolysis, (2) previous operations due to a herniated disc, joint preservation, or alloplastic surgery at the hip, knee, or ankle region, and (3) neurological or rheumatological diseases.

**Figure.**
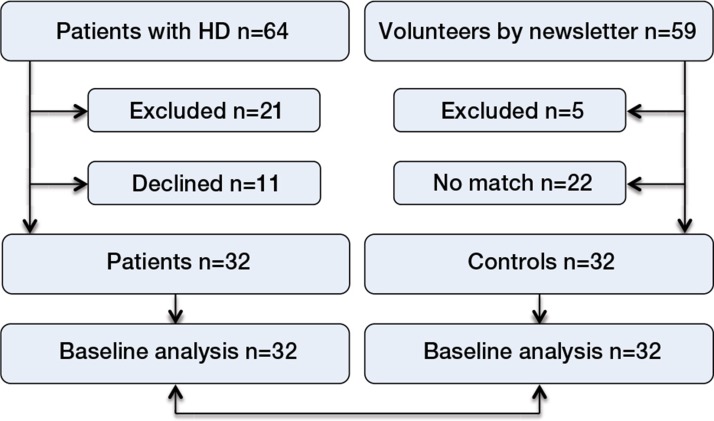
32 patients with hip dysplasia were included from the Division of Hip Surgery at the Department of Orthopedics at Aarhus University Hospital in Denmark from March 1st 2011 to December 1st 2011.

Parallel to the inclusion of patients, a control group of 32 individuals with no hip, knee, ankle, or back problems were included from the patients’ social network and through the hospital’s intranet. The number of participants was based on an a priori sample-size calculations for net joint moment of hip flexion and hip extension angle, which were based on previous studies evaluating the gait pattern in patients with hip dysplasia (Romano et al. 1996, Pedersen et al. 2004); we found that a minimum of 22 participants in each group would be needed in the present study (based on a power of 80%).

### Design and procedure

Peak joint angles and peak net joint moments of the lower extremity were compared between the groups, and the 32 patients were frequency-matched with the control group based on sex and age (± 5 years). Baseline characteristics were registered using standardized questions. Pain was measured on a 100-mm visual analog scale (VAS). Wiberg’s center-edge (CE) angle, Tönnis’ acetabular index (AI) angle, and osteoarthritis grade were measured on anteroposterior radiographs, while information from the hospital charts was used to record unilateral or bilateral involvement and other pathologies. Clinical or radiographic examinations were not conducted on the healthy controls, and none reported present or previous problems with their legs or back. All participants completed the HAGOS questionnaire at the same time as the walking and running analyses. HAGOS has been validated to measure the status of health in young to middle-aged physically active patients with longstanding hip and/or groin pain. HAGOS has been found to have good test-retest reliability, with a smallest detectable change (SDC) of between 2.7 points and 5.2 points out of 100 points at the group level (Thorborg et al. 2011).

Apart from age, sex, BMI, and limb dominance, we recorded unilateral/bilateral involvement, congenital hip dislocation, osteoarthritis grade, and CE and AI angles to describe the severity of hip dysplasia in the patient group. Data on duration of pain prior to surgery and intake of analgesia were recorded. This is the first part of a prospective study where changes in analgesia consumption over time will be evaluated.

### Data acquisition

The primary outcomes were the peak net joint moment of hip flexion during the second half of stance and the peak hip extension angle during stance. A motion-capture recording was done at the Department of Sport Science, Aarhus University, Denmark, where the participants walked and ran at self-selected speeds along an 8-m walkway. They walked with bare feet and ran with neutral running shoes (New Balance NB-W 759 PS; New Balance; Maine, USA). Kinematic data were recorded at 240 Hz with an 8-camera ProReflex MCU 1000 motion-capture system (Qualisys AB, Gothenburg, Sweden). Ground reaction forces were simultaneously sampled at 960 Hz using an OR6-7 AMTI force plate (Advanced Mechanical Technology, Watertown, MA). During walking and running, participants were equipped with thirteen 19-mm reflective markers on each limb, placed according to the Visual3D conventional marker set guidelines (C-Motion Inc., Germantown, MD) (Cappozzo et al. 1997, Robertson et al. 2004). Briefly, 8 markers were placed on 2 lightweight rigid plates on each limb, so-called clusters (McClay and Manal 1999, Manal et al. 2000), at the thigh and shank, while the remaining 5 were placed at the spina iliaca anterior superior, spina iliaca posterior superior, calcaneus, caput of the first metatarsal and caput of the fifth metatarsal. These markers allow each segment of the limb (foot, shank, and thigh) and the pelvis to be treated as a 6-degrees-of-freedom rigid segment. In addition to the walking and running recordings, a static recording with 7 extra markers on crista iliaca, trochantor major, condylus medialus et lateralis, malleolus medialis et lateralis, and calcaneus was performed, allowing calculation of the positions of the ankle, knee, and hip joint centers relative to the 4 rigid segments.

The anatomical landmarks where the non-cluster markers were placed were palpated by the same experienced investigator (DBN) according to Van Sint Jan (2007).

The camera system was calibrated before the recordings, producing residual errors of less than 2.5 mm over a volume of approximately 3 m × 1 m × 1.3 m (L × W × H). At least 3 right and 3 left dynamic trials were recorded, where the participant had to hit the force plate with the whole foot and where the walking speed was stable.

Before testing, the participants rated pain at rest on a 100-mm VAS scale, and immediately after testing they rated pain during activity.

### Data processing

2-D marker-position data from each of the 8 cameras were combined into a 3-D representation using the Qualisys Tracking Manager software (Qualisys AB, Gothenburg, Sweden). The marker-position and force-plate data were then exported to Visual3D (C-Motion Inc.) for further analysis. The data were low-pass filtered with a fourth-order, zero-lag Butterworth filter with a cutoff value of 6 Hz for walking and 12 Hz for running for the marker-position data, and 30 Hz and 45 Hz, respectively, for the force-plate data. The filtered data were subsequently used together with anthropometric data, calculated from individual body mass and height using Dempster’s equations (Dempster 1955) as input for an inverse-dynamics calculation resulting in sagittal joint angular positions and net joint moments of the hip, knee, and ankle in the stance phase. Angles were calculated based on the Joint Coordinate System proposed by Grood and Suntay (1983), and 0 degrees at the joints corresponded to an erect standing position. Hip flexion, knee extension, and ankle dorsal flexion were assigned as positive values. To identify changes between the patients and healthy controls, peak values of the joint angles and net joint moments were tested statistically. Right or left trials were selected for the statistical analysis, based on the affected limpb of the patient. In patients with bilateral involvement, the trials for the limb undergoing operation were selected. Trials corresponding to same limb in the matched controls were selected for analysis.

### Statistics

The distribution of data was assessed with scatter plots and histograms. Normally distributed data are presented as mean (SD); otherwise, the data are presented as median (range). In the normally distributed data, Student’s t-test was used to evaluate differences between the groups, and a multiple regression analysis was performed to adjust for possible variation in age and sex in the mechanical outcomes, because earlier studies have suggested that age and sex affect kinematics and kinetics of walking (Bohannon 1997, DeVita and Hortobagyi 2000, Stoquart et al. 2008). Using Spearman’s correlation, we finally tested whether the primary outcomes correlated to the HAGOS subscales pain and sport/recreation. Differences between the groups are presented with 95% confidence interval (CI) and the primary outcomes peak hip extension and peak net joint moment of hip flexion are presented with an α level of 0.05 but were tested statistically at a level of 0.025 (Bonferroni correction).

### Ethics

The study followed the tenets of the Helsinki Declaration of 1975, and all participants consented to participate in the study. The Central Denmark Region Committees on Biomedical Research Ethics approved the study on September 30, 2010 (M-20100206). The Danish Data Protection Agency gave permission for the handling of personal data, and the study was registered at ClinicalTrials.gov (NCT01344421).

## Results

### Baseline characteristics (Table 1)

The statistical analysis did not reveal any differences in the duration of the stance phase in either walking or running between groups based on a power of 80%. The mean duration of the stance phase in walking was 0.62 (SD 0.06) s in the patients and 0.60 (SD 0.03) s in the controls. In running, the mean values were 0.31 (SD 0.05) s in the patients and 0.30 (SD 0.04) s in the controls. In contrast, the mean velocity during walking was lower in the patients than in the controls (1.31 (SD 0.19) m/s as opposed to 1.41 (SD 0.14) m/s; p = 0.02). This was not the case for running (2.46 (SD 0.43) m/s vs. 2.57 (SD 0.34) m/s; p = 0.3). In all dimensions of HAGOS, the patients reported lower scores than the controls.

**Table 1.** Baseline characteristics ^a^ of the patients and healthy controls

Outcomes	Patients	Controls	p-value
			
Women, n	26	26	–
Age, years (range)	34 (18–53)	33 (18–54)	–
Limb dominance, right n	29	31	0.6
BMI (range)	22 (15–29)	22 (16–31)	0.7
Bilateral/unilateral n	24/8	–	–
Congenital hip dislocation, n	4	–	–
Duration of pain 0.0–5.0 years, n	19	–	–
Duration of pain 5.1–10.0 years, n	9	–	–
Duration of pain > 10 years, n	4	–	–
Non-prescription analgesia, n	6	–	–
Prescription analgesia, n	8	–	–
Osteoarthritis grade 0/grade 1, n	26/6	–	–
CE angle preoperatively (range)	18 (4–22)	–	–
AI angle preoperatively (range)	14 (10–22)	–	–
HAGOS pain, 0–100	49 (20–95)	100 (85–100)	< 0.001
HAGOS symptoms, 0–100	46 (21–96)	96 (79–100)	< 0.001
HAGOS ADL, 0–100	58 (5–100)	100 (85–100)	< 0.001
HAGOS sport/recreation, 0–100	36 (3–91)	100 (84–100)	< 0.001
HAGOS participation, 0–100	25 (0–100)	100 (50–100)	< 0.001
HAGOS quality of life, 0–100	38 (0–80)	100 (75–100)	< 0.001

**^a^** Baseline characteristics are presented as median values (range) and as numbers for the patients and the healthy controls. Differences between the groups were tested with Wilcoxon signed-rank test and Fisher’s exact test. BMI: body mass index; HAGOS: Copenhagen Hip and Groin Outcome Score; ADL: activities of daily living; CE (center-edge); AI: Tönnis’ acetabular index.

### Walking and running analysis (Tables 2 and 3)

In walking, the peak hip extension angle in stance was significantly lower in the patients than in the controls, and the peak net joint moment of hip flexion in the second half of stance was significantly lower in the patients than in the controls. In running, the patients tended to have a lower peak net joint moment of hip flexion. In both walking and running, pain was reported to be higher by the patients (p < 0.001). The patients reported a median VAS value at rest of 12 (0–77) mm; for walking, they reported a median VAS value of 9 (0–83) mm, and for running the median VAS value was 18 (0–97) mm. The healthy controls reported a median VAS value at rest, during walking, and during running of 0 (0–11) mm. Adjusted analyses were performed to compensate for a possible variation in age and sex in the mechanical outcomes, but the analyses did not reveal any relevant changes in the kinematic and kinetic data.

**Table 2.** Peak joint angles in patients and healthy controls

Peak	Patients **^a^**	Controls **^a^**	Difference (95% CI)	p-value
	(n = 32)	(n = 32)		
*Walking (°)*				
Ankle				
A1	–8.9 (2.1)	–9.6 (2.4)	–0.7 (–1.8 to 0.4)	0.2
A2	8.8 (3.5)	7.3 (2.8)	–1.5 (–3.1 to 0.1)	0.07
Knee				
K1	–4.1 (3.8)	–4.5 (5.1)	–0.4 (–2.7 to 1.8)	0.7
K2	–16 (3.7)	–18 (5.5)	–1.6 (–3.9 to 0.8)	0.2
K3	–5.5 (4.3)	–3.0 (4.4)	2.5 (0.3 to 4.7)	0.03
Hip				
H1	–10 (4.8)	–13 (4.5)	–2.7 (–5.1 to –0.4)	0.02
*Running (°)*				
Ankle				
A1	1.2 (5.5)	2.2 (3.8)	0.2 (–2.2 to 2.9)	0.9
A2	20 (4.5)	21 (3.8)	1.4 (–0.7 to 3.5)	0.2
Knee				
K1	–13 (6.2)	–15(6.6)	–2.0 (–5.2 to 1.2)	0.2
K2	–39 (7.6)	–42 (5.8)	–2.7 (–6.0 to 0.7)	0.1
K3	–16 (6.1)	-15 (5.6)	1.3 (–1.7 to 4.2)	0.4
Hip				
H1	–4.0 (5.1)	–5.6 (3.9)	–1.6 (–3.8 to 0.7)	0.2

**^a^** Values are mean (SD).

**Table 3.** Peak net joint moments in patients and healthy controls

Peak	Patients **^a^**	Controls **^a^**	Difference (95% CI)	p-value
	(n = 32)	(n = 32)		
Walking (N*m/kg)				
Ankle				
MA1	0.18 (0.05)	0.20 (0.05)	0.01 (–0.01 to 0.04)	0.3
MA2	–1.48 (0.17)	–1.56 (0.17)	–0.07 (–0.16 to 0.01)	0.08
Knee				
MK1	–0.42 (0.17)	–0.46 (0.11)	–0.04 (–0.11 to 0.03)	0.2
MK2	0.36 (0.17)	0.46 (0.19)	0.10 (0.01 to 0.19)	0.04
MK3	–0.42 (0.17)	–0.54 (0.14)	–0.12 (–0.20 to –0.04)	0.003
MK4	0.21 (0.05)	0.26 (0.13)	0.04 (0.01 to 0.09)	0.08
Hip				
MH1	–1.08 (0.34)	–1.19 (0.20)	–0.11 (–0.25 to 0.03)	0.1
MH2	0.57 (0.13)	0.70 (0.22)	0.13 (0.04 to 0.22)	0.008
Running (N*m/kg)				
Ankle				
MA1	0.15 (0.16)	0.17 (0.12)	0.02 (–0.05 to 0.09)	0.6
MA2	–2.27 (0.45)	–2.32 (0.41)	–0.04 (–0.26 to 0.17)	0.7
Knee				
MK1	–0.50 (0.20)	–0.49 (0.18)	–0.01 (–0.09 to 0.10)	0.9
MK2	1.64 (0.48)	1.87 (0.42)	0.23 (0.01 to 0.46)	0.04
MK3	–0.29 (0.29)	–0.20 (0.15)	0.09 (–0.03 to 0.20)	0.2
MK4	1.29 (0.49)	1.58 (0.41)	0.29 (0.07 to 0.52)	0.01
Hip				
MH1	–1.57 (0.46)	–1.74 (0.42)	–0.17 (–0.39 to 0.05)	0.1
MH2	0.65 (0.42)	0.85 (0.30)	0.20 (0.01 to 0.38)	0.04

**^a^** Values are mean (SD).

### Correlations between HAGOS and outcomes of walking and running (Table 4)

Statistically significant correlation coefficients were found for both mechanical outcomes in walking and the HAGOS subscales. In running, there was only a tendency of a correlation between the peak net joint moment of hip flexion and the subscale sport/recreation. Furthermore, a subanalysis showed that there were significant correlations between the HAGOS subscales (r = 0.96, p < 0.001).

**Table 4.** Correlations ^a^ between the mechanical outcomes and HAGOS

	HAGOS pain		HAGOS sport/recreation	
Baseline	Spearman’s rho	p-value	Spearman’s rho	p-value
H1 walking	–0.30	0.02	–0.29	0.02
MH2 walking	0.34	0.007	0.34	0.007
H1 running	–0.17	0.2	–0.15	0.3
MH2 running	0.23	0.07	0.26	0.04

**^a^** Correlations between the peak hip extension angle (H1) and the HAGOS subscales pain and sport/recreation. Also, correlations between the peak net joint moment of hip flexion (MH2) and the HAGOS subscales pain and sport/recreation.

## Discussion

As hypothesized, the patients had a significantly lower peak hip extension angle and a lower peak net joint moment of hip flexion during walking than the healthy controls. Furthermore, in walking both the peak hip extension angle and the net joint moment of hip flexion correlated with the HAGOS subscales pain and sport/recreation. In running, both the net joint moment of hip flexion and the hip extension angle were lower in the patients than in the controls but not statistically significantly so.

Hip flexor muscles form the net joint moment of hip flexion together with the joint capsule and the strong capsule ligaments. The net joint moment is formed at the end of the stance phase and reduces hip extension by accelerating the limb forward. A deficit of the hip flexors may explain the reduced peak net joint moment of hip flexion, while pain in the hip flexors or a deficit in the hip extensors may explain the reduced peak hip extension angle.

Previous studies have reported deficits in walking in patients with hip dysplasia. Pedersen et al. (2004) reported a statistically significant lower peak net joint moment of hip flexion in 14 women with hip dysplasia than in 12 healthy women, but this did not apply to peak hip extension. This could have been due to small sample size. Romano et al. (1996) also reported kinematic and kinetic walking outcomes in patients with unilateral hip dysplasia, and found reduced peak external hip flexor moments compared to healthy controls. Sucato et al. (2010) evaluated walking in patients with bilateral symptomatic hip dysplasia and reported lower walking speed in the patients than in healthy controls. Overall, we found the same differences for the peak net joint moment of hip flexion, the peak hip extension angle, and for walking speed as reported in the previous studies, but the differences in running in our study were not statistically significant, perhaps due to the fact that we Bonferroni-corrected our level of significance, in contrast to the studies by Pedersen et al. (2004) and Romano et al. (1996). Our use of the Bonferroni correction may be interpreted as a conservative approach. It was done in order to minimize the risk of type-1 error, because our primary outcomes were neither associated nor unassociated—but somewhere in-between. We did not find any significant differences for peak hip extension angle and peak net joint moment of hip flexion during running, and Pedersen at al. (2004) did not find any significant difference for the peak hip extension angle during walking. However, our study and the previous studies in general found reduced peak joint angles in hip extension and reduced peak net joint moments of hip flexion (Romano et al. 1996, Pedersen et al. 2004). This consistency indicates that the differences found in the previous studies and in the present study do indeed exist, and the lack of statistical significance can probably be explained by sample sizes that were too small.

Even though the design of our study was simple and although we applied methods that are commonly used, there were limitations. Firstly, the healthy controls did not undergo radiographic or clinical examination of the hip before inclusion, and although the risk was small, we cannot rule out that some of the healthy controls may have had undiagnosed asymptomatic hip dysplasia, since this condition is prevalent in approximately 4% of the Danish population (Jacobsen et al. 2005, Gosvig et al. 2010). However, this would only have resulted in smaller differences between the groups and would not have led to overestimation of our results.

Secondly and more importantly, the participants were walking and running at a self-selected speed; thus, an effect of this may have been that the differences in peak hip extension and in peak net joint moment of hip flexion between the groups were a result of differences in speed and not differences in walking and running. Stoquart et al. (2008) reported that kinematic and kinetic walking variables increase as a result of a higher gait speed. This is in line with the work of Oberg et al. (1994), who reported that gender and speed affect the walking pattern. However, Pedersen et al. (2004) reported differences for peak hip extension angle and peak net joint moment of hip flexion during walking with a speed of 4.5 km/h in both patients and controls. We found similar differences in peak hip extension and peak net joint moment of hip flexion in running, and there were no differences in speed between the patients and the controls in our study. This consistency indicates that symptomatic hip dysplasia affects the walking and running patterns, and that the differences we found were not a result of differences in speed. Furthermore, a specific speed may not represent a normal, spontaneous movement pattern.

A third limitation was that only sagittal-plane outcomes were evaluated. We chose to evaluate the sagittal plane as in the studies by Pedersen et al. (2004, 2006). We judged that sagittal-plane evaluation of movement ought to be sufficient since Sucato et al. (2010) found no differences in the frontal plane kinetics in walking in a similar group of patients. However, Romano et al. (1996) reported differences in the frontal and transversal planes, and we cannot rule out the possibility that differences existed in these planes in our patients.

A fourth limitation was the fact that we did not find statistically significant differences between the groups in running. One reason—despite the fact that we made a sample-size calculation—was that the standard deviations in running turned out to be larger than expected. This was probably due to different running patterns, as for an example the heel-toe footfall pattern or the flat-foot pattern (Robertson et al. 2004). Another reason could be that the stance phase in running takes up a smaller percentage of the running cycle than in walking, resulting in differences in the timing of the individual joint angles and net joint moments in running compared to walking (Robertson et al. 2004). Also, the magnitudes of the net joint moments are different because running is a more forceful activity (Robertson et al. 2004). Finally, the participants were running at a pace that resulted in smaller hip joint angles compared to walking, which could explain why no differences were found in running. The VAS values in walking and running were similar, and therefore pain cannot explain why no significant differences were found in running. However, significant differences were found in walking, indicating that evaluations of the hip extension angle and the net joint moment of hip flexion in walking are sufficient and relevant if surgical or training interventions for patients with hip dysplasia are to be evaluated in future studies.

Despite the limitations discussed above, the present study showed substantial walking deficits in patients with symptomatic hip dysplasia, and there were correlations between walking and self-reported pain and sport/recreation. This indicates that the kinematic and kinetic deficits follow self-reported pain and sport/recreation, and even though the correlation might be interpreted as weak, the differences in walking and running that we found are clinically relevant. However, the relatively weak correlation indicates that movement as measured in a movement analysis and self-reported health status measure different aspects of the impact of hip dysplasia.

One of our reasons for including running in this study was to evaluate an activity that is more demanding than walking in these rather young dysplasia patients, but even though there were deviations in running relative to the controls, evaluation of walking seems to be adequate in these patients.

We will follow these patients to investigate whether the minimally invasive transsartorial approach that we use for periacetabular osteotomy will normalize the peak hip extension angle and peak net joint moment of hip flexion at 6 months and 12 months after the operation, and if self-reported pain and health status improve as a result of the surgical intervention. Future studies should focus on evaluating the role of pelvic and trunk motion together with frontal and transversal plane kinetics and kinematics.
